# Ovarian Hyperstimulation Syndrome, the Master of Disguise?

**DOI:** 10.1155/2015/510815

**Published:** 2015-02-22

**Authors:** Emily Charlotte Ironside, Andrew James Hotchen

**Affiliations:** Oxford University Hospitals, Headley Way, Headington, Oxford OX3 9DU, UK

## Abstract

The use of IVF has risen dramatically over the past 10 years and with this the complications of such treatments have also risen. One such complication is ovarian hyperstimulation syndrome with which patients can present acutely to hospital with shortness of breath. On admission, a series of blood tests are routinely performed, including the d-dimer. We present a case of a 41-year-old lady who had recently undergone IVF and presented with chest pain and dyspnoea. In the emergency department, a d-dimer returned as mildly elevated. Consequential admission onto MAU initiated several avoidable investigations for venous thromboembolism. Careful examination elicited a mild ascites and a thorough drug history gave recent low molecular weight heparin usage. Ultrasound scan of the abdomen subsequently confirmed the diagnosis of severe OHSS. The d-dimer should therefore be used to negate and not to substantiate a diagnosis of VTE. This case report aims to highlight the importance of OHSS as an uncommon cause of dyspnoea but whose prevalence is likely to increase in the forthcoming years. We discuss the complications of the misdiagnosis of OHSS, the physiology behind raised d-dimers, and the potential harm from incorrect treatment or inappropriate imaging.

## 1. Introduction

Ovarian hyperstimulation syndrome (OHSS) is a well-recognized iatrogenic complication of assisted conception techniques, including* in vitro* fertilization (IVF) [1]. Although the majority of presentations are mild, severe cases can result in systemic capillary leakage, causing life-threatening complications such as thromboembolic phenomena and multiple organ dysfunctions [2].

OHSS is common, occurring in mild forms in 33% of IVF cycles and in moderate or severe forms in 3% to 8% of IVF cycles [3]. Although it can occur in all age groups, it is less common in women over the age of 39 years [4]. In the last 10 years, in the United States, there has been a 50% increase in the number of IVF treatments in women over 41 years of age [5]. OHSS is particularly topical following a recent update of guidelines in the United Kingdom, which extends the age of those who can receive treatment to 42 years [6]. This recent increase in the usage of IVF will inevitably result in a rise in the number of cases of OHSS seen in the emergency department (ED). Ultimately, this will give the emergency physician an important role in expediting and optimizing treatment for these patients. On admission to the ED, a plethora of blood investigations are requested for those who present with acute shortness of breath including complete blood count, urea and electrolytes, troponin, and a d-dimer. The results of these investigations need to be interpreted with care as misinterpretation can lead to serious consequences for the patient and a delay in treatment.

We report a case of OHSS that was initially misdiagnosed in the ED, attributable to a mildly raised d-dimer, resulting in transfer to the inappropriate specialty and incorrect treatment being commenced. We discuss the potential complications for misdiagnosis of OHSS and the pathophysiology behind the raised d-dimer. This case report highlights an important message for the emergency physician and raises awareness of this increasingly common iatrogenic condition.

## 2. Case Report

A 41-year-old woman, undergoing her second cycle of IVF treatment, presented to the ED with acute chest pain. The chest pain was central, was worse on inspiration, and was not induced or exacerbated by exercise. The patient had associated dyspnea and observations revealed oxygen saturations to be 90% on air. Her thrombogenic risk factors included reduced immobility due to back pain and recent IVF [7]. Past medical history included one failed cycle of IVF and a recent embryo implantation in her second IVF cycle. Examination revealed a woman in substantial pain, with associated tachypnea, tachycardia, and bilateral reduction in air entry to the lung bases.

The presence of thrombogenic risk factors and the clinical presentation gave the patient a modified Wells score of six, rendering pulmonary embolus a likely diagnosis [8]. Consequently, the patient was placed on high flow oxygen and routine bloods, a d-dimer, and a clotting profile were requested. The d-dimer returned as mildly raised (430 mg/L, upper limit of normal in our laboratory was 250 mg/L) and the patient was sent to the medical admissions unit (MAU) for further clerking and therapeutic thromboembolic treatment.

In the MAU, two important inconsistencies with the original assessment were established. Firstly, the chest pain appeared to be epigastric and was associated with new onset abdominal bloating. Secondly, a thorough drug history highlighted that the patient had been taking a low molecular weight heparin following her previous IVF failure. These findings significantly reduced the likelihood of a pulmonary embolus. Consequently, beta-human chorionic gonadotropin (beta-hCG) levels and abdominal ultrasound scan were requested. The beta-hCG returned as raised and the scan revealed bilateral ovarian enlargement, ascites, and a right-sided pleural effusion, uniting the symptoms and thus confirming the diagnosis of severe OHSS.

Subsequently, the patient was transferred to gynecology where she was closely monitored. During her stay, the ascites reduced, the shortness of breath improved and the following week, she was discharged home with no symptoms.

## 3. Discussion

This case has illustrated a diagnosis that is important in patients who are of reproductive age. We have reported a case of severe OHSS which was mistaken for thromboembolic disease due to an inadequate history combined with a reliance on blood tests that could have potentially led to serious complications.

The complications of OHSS depend on the severity of the condition although a misdiagnosis and mistreatment can potentially become fatal. Complications from mild cases are usually self-limiting. In the more severe forms, fluid shifts can lead to dehydration resulting in acute kidney injury, multiple organ failure, and adult respiratory distress syndrome. Dehydration also increases the risk of thromboembolic phenomena and this occurs in 0.7% to 10% of OHSS patients [9]. Thromboembolic disease is therefore an important condition to rule out in any potential patient who has had assisted reproductive technologies (ART). This promotes the rationale behind the referral of our patient to the MAU with a suspected pulmonary embolus. However, it also highlights the importance of taking a thorough medication history, which revealed that the patient had recently been started on a low-molecular weight heparin in addition to thromboembolic stockings, two factors that reduce the risk of a thromboembolic disease.

**Figure 1:**
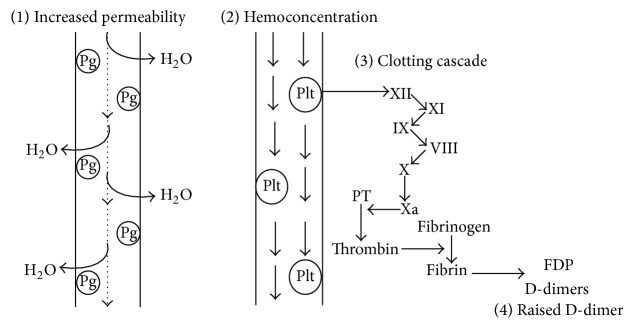
Schematic illustration of how OHSS causes a rise in d-dimers. Pg = prostaglandin; Plt = platelets; FDP = fibrin degradation productions; PT = prothrombin. (1) Dotted arrow represents normal blood flow. Prostaglandins are increased in OHSS which causes increased capillary permeability. This forces water out of the capillaries and into the tissues. (2) Loss of water causes hemoconcentration of the blood which is represented by thick arrows. Due to this, activation of platelets occurs. (3) Activation of platelets causes activation of the clotting cascade (simplified without the activated factors). (4) The final products of the coagulation cascade are fibrin degradation products which can be measured in the blood as d-dimers.

In OHSS, the pathophysiology behind the raised d-dimer is thought to be due to an elevation of prostaglandins, which increase vascular permeability and result in extravasation of fluids into the third space. Extravasation leads to hemoconcentration, which in turn increases serum viscosity and slows blood flow. The hematological changes increase endothelial adherence of platelets and activate the coagulation cascade. In order to prevent the formation of thrombi, the body generates endogenous hormones to dissolve the fibrin clot. This ultimately increases fibrin degradation products which are measured as the d-dimer [10]. This whole process is illustrated in Figure 1.

This case imparts an important lesson regarding the interpretation of the investigations performed in the ED, especially the d-dimer. D-dimers are fibrin degradation products, which have a high sensitivity but low specificity. They can be elevated in a plethora of conditions including infection, inflammatory disease, malignancy, OHSS, and pregnancy [11]. In this case, the d-dimer was used to substantiate the diagnosis of a thromboembolic disease. Acting on a raised d-dimer is of particular significance as radiological investigations, which are often required for diagnoses of emboli, could be harmful to both the expectant mother and her fetus [12]. This supports the use of d-dimers only to rule out a pulmonary embolus and not to substantiate the history and clinical findings. The case also highlights that there is a relationship between thromboembolic disease and OHSS and that both conditions need to be considered when treated patients have undergone ART. This needs to be highlighted so that vital treatments are not omitted with potentially life threatening complications.

## 4. Why Should an Emergency Physician Be Aware of This?

Shortness of breath is a common presenting complaint to the ED. For this, it is important to consider multiple etiologies for abnormal blood results, especially d-dimers. D-dimer testing is useful only for negating and not substantiating a diagnosis of pulmonary embolism. This case report aims to highlight the importance of OHSS as an uncommon cause of dyspnea, but whose prevalence is likely to increase in the forthcoming years as a number of ART procedures are performed.

## References

[1] Klemetti R., Sevón T., Gissler M., Hemminki E. (2005). Complications of IVF and ovulation induction. *Human Reproduction*.

[2] Stewart J. A., Hamilton P. J., Murdoch A. P. (1997). Thromboembolic disease associated with ovarian stimulation and assisted conception techniques. *Human Reproduction*.

[3] Delvinge A., Rozenberg S. (2002). Epidemiology and prevention of ovarian hyperstimulation syndrome (OHSS): a review. *Human Reproduction Update*.

[4] Bancsi L. F. J. M. M., Broekmans F. J. M., Eijkemans M. J. C., de Jong F. H., Habbema J. D. F., Te Velde E. R. (2002). Predictors of poor ovarian response in in vitro fertilization: a prospective study comparing basal markers of ovarian reserve. *Fertility and Sterility*.

[5] SartCors Clinical Summary Report. https://www.sartcorsonline.com/.

[6] NICE (2013). *Fertility: Assessment and Treatment or People with Fertility Problems. Clinical Guidelines*.

[7] Aurousseau M. H., Samama M. M., Belhassen A., Herve F., Hugues J. N. (1995). Risk of thromboembolism in relation to an in-vitro fertilization programme: three case reports. *Human Reproduction*.

[8] Wells P. S., Ginsberg J. S., Anderson D. R. (1998). Use of a clinical model for safe management of patients with suspected pulmonary embolism. *Annals of Internal Medicine*.

[9] Delvigne A., Rozenberg S. (2003). Review of clinical course and treatment of ovarian hyperstimulation syndrome (OHSS). *Human Reproduction Update*.

[10] Alper M. M., Smith L. P., Sills E. S. (2009). Ovarian hyperstimulation syndrome: current views on pathophysiology, risk factors, prevention, and management. *Journal of Experimental and Clinical Assisted Reproduction*.

[11] Lip G. Y. H., Lowe G. D. (1995). Fibrin D-dimer: a useful clinical marker of thrombogenesis?. *Clinical Science*.

[12] Moradi M. (2013). Pulmonary thromboembolism in pregnancy: diagnostic imaging and related consideration. *Journal of Research in Medical Sciences*.

